# The promiscuous activity of alpha-amylase in biodegradation of low-density polyethylene in a polymer-starch blend

**DOI:** 10.1038/s41598-019-39366-0

**Published:** 2019-02-22

**Authors:** M. Karimi, D. Biria

**Affiliations:** Department of Biotechnology, Faculty of Advanced Sciences and Technologies, University of Isfahan, Isfahan, Iran

## Abstract

Blending polyolefins with certain types of natural polymers like starch can be beneficial to their biodegradation. The impact of alpha-amylase on the biodegradation of low-density polyethylene (LDPE)-starch blend samples in an aqueous solution was investigated through characterizing their physical, mechanical and chemical properties. Results indicated that the weight and tensile strength of the enzyme treated samples were reduced by 48% and 87% respectively. Moreover, differential scanning calorimetry (DSC) showed an increase in fusion enthalpy of degraded samples which means that the crystallinity has been increased. The biodegradation of LLDPE appeared in Fourier-transform infrared spectroscopy (FT-IR) through the reduction in the intensity of the related peaks. This observation was supported by energy dispersive x-ray spectroscopy (EDXS) analysis where decreasing the percentage of carbon atoms in the treated blend was obtained. Likewise, the gel permeation chromatography (GPC) results pointed to a significant reduction in both the molecular weight and viscosity of LDPE more than 70% and 60% respectively. Furthermore, thermal gravimetric analysis (TGA) affirmed the function of amylase in degradation of the blend. On the basis of the obtained results, it can be claimed that the main backbone of the polymer, as well as the side branches, have been scissored by the enzyme activity. In other words, alpha-amylase has a promiscuous cometabolic effect on biodegradation of LDPE in polymer-starch blends.

## Introduction

Polyolefins have been extensively utilized in a wide range of industrial and domestic applications. They have many advantages such as suitable chemical and physical properties, low prices, high capacity of production, and well-established facilities and processing methods which support their increasing usage trend in the coming years. However, the recalcitrant nature of these petroleum-based polymers makes them a threat to the environment. Although the biodegradation of polyolefins is feasible in the natural environment, it can prolong to a few centuries. As a remedy, several solutions such as photodegradation, thermal degradation, and addition of degradation accelerators (e.g., pro-oxidant) have been suggested to enhance their biodegradability^1,2^. In addition, it has been reported that blending of the polyolefins with certain types of natural polymers such as the starch can be beneficial to their biodegradation^3–5^. The specific characteristics of the starch in combination with the biodegradability lead to be considered as an alternative to the petroleum-based plastics in the form of composites and blends with the proper materials^6^. In fact, owing to the properties such as the gel formation, adhesion, swelling, physical and chemical transformation capabilities, starch has been suggested as a principal part of several polymeric mixtures to find applications in the medical, food and scientific domains^7,8^.

The weight and mechanical properties of the starch blended polymers were reported to be lower than the blank samples after treatment with microorganisms^9^. The suggested mechanism for the biodegradation improvement has been related to the fast hydrolysis of starch which makes the polymer porous and more susceptible to both biotic and abiotic degradations^10–12^. Consequently, the changes in the physical structure and crystallinity of the polymer will affect its mechanical properties^13^. Furthermore, it has been reported that starch has a synergetic effect on UV photo-oxidative degradation of PE through enhancing the production of carbonyl groups in the polymer structure^12^. However, reports on biodegradation of PE-starch blends indicated that with the exception of the presence of pro-oxidants in the system, the molar mass of polyethylene was nearly unchanged even though the physical and mechanical properties of the samples have decreased significantly^14–17^. Although the presented evidence in the literature supports the suggested mechanisms, the above explanations for polyolefins biodegradation improvement seem to be incomplete^1,18^. It has been reported that the accessibility of starch in its blend with a polymer has been greatly limited especially in lower starch contributions (i.e., less than 30%) which are quite usual in preparing the blends^19^. In fact, most of the presented scanning electron microscopy (SEM) pictures of the starch polymer blends after the degradation process have shown a few superficial tiny cracks and pores which cannot affect the available surface area of the polymer significantly^9,12^. A fine powder of the polymer would be more susceptible to degradation if the surface area were helpful for PE degradation. On the other hand, considering the production of active moieties and free radicals as the driving force of degradation is hardly satisfactory because the free radicals react through a fast propagating mechanism and the polymer should have demolished in a short time^20^.

In fact, the primary target of blending PE with starch is elevating the degradation of polyethylene in the prone conditions. The starch is well-known as a biodegradable polymer and in almost all of the published researches its degradation in the blend has been reported. Therefore, it is rational to think that there must be something about the degradation of starch affecting the PE breakdown. Alpha-amylase is a specific enzyme for starch hydrolysis, and the starch biodegradation has been mainly referred to its activity. Consequently, it can be considered as an effective factor in the PE degradation. Recently, it has been reported that amylase can be effective on the degradation of saturated linear hydrocarbons^21^. Accordingly, the similarity between the chemical structure of paraffin hydrocarbons and polyethylene can inspire that the biodegradation of polyethylene is likely to be enhanced in the presence of amylase in the same way. In fact, the possible chemical decomposition of the polyethylene molecules at the presence of starch and amylase can lead to decrease in the molecular weight of the polymer. Besides, a blend of starch with a hydrophobic polymer like PE can be stabilized through the inclusion mechanism with the V-type conformation of the starch molecules^22–24^. The partial decomposition of starch in such a blend can unbalance the hydrophobic-hydrophilic interactions which cause cracks in the physical structure of the system. In this work, an attempt is made to investigate the above-mentioned mechanisms for degradation of a starch-PE blend in the presence of alpha-amylase.

## Result and Discussions

### Physical changes

The Physical changes in the body of samples can be considered as the first clue of degradation. In fact, decomposition of materials in a degradation process accompanies with apparent deficiencies in the shape of the sample. The physical appearance of the blank and treated samples has been shown in Fig. 1s of Supplementary Materials. The samples treated with the enzyme solution exhibit edge-delamination, decay, and flake. In contrast, the blank samples (incubated in water without enzyme) have mainly preserved their original physical structure. These results demonstrated that the addition of hydrophilic starch into hydrophobic polyethylene propagates the hydrophilicity and biodegradability of the polymer blend^25^. The observed biodegradation here has been studied in more details in the following sections.

### Surface microstructure

**Figure 1:**
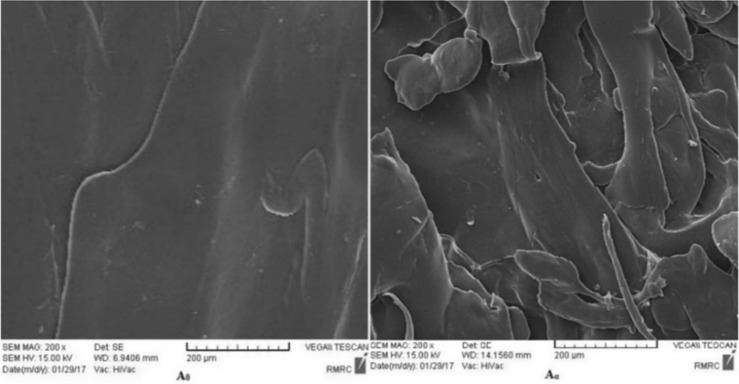
Scanning electron micrographs of PE-starch (A_0_) before and (A_α_) after enzyme treatment.

It is reported that the native starch has an A-type semi-crystalline structure while the thermoplastic starch forms the V-type crystalline conformation^26,27^. The A-type starch can be converted to the V-type through plasticization by a plasticizer and implementation of shear force in an extruding process^6^. Previous studies which have reported scanning electron micrographs for the native starch shown spherical and oval granules in various sizes, without craze line, crack, pores and fissure while the thermoplastic starch micrographs shown a smooth surface^28,29^. It has been suggested that in the plasticization of starch, H-bonds’ failure between the starch molecules and the formation of H-bonds between starch and plasticizer molecules (e.g. glycerol) can simultaneously occur^30^. Moreover, an increase in the percentage of glycerol leads to increasing the average intermolecular distance by swelling^31^. Therefore, the plasticization effect obtained by both the addition of glycerol (as the plasticizer) and the shear forces in the samples producing procedure in this work reduces the interactions between the starch chains and simplifies their movement^32^. The homogenous surface without any granules which has been taken by scanning electron microscopy (SEM) (Fig. 1, sample A_0_) supports the idea that the starch has been formed in the V-type structure in the blend with PE. Moreover, the partial hydrolysis of the starch in the blend with PE due to the effect of amylase has caused loss of the sample integrity by the formation of some physical defects such as cavities, pinholes, and non-level multiple layers. Fig. 1 (A_α_) displays the surface of a PE-starch sample subjected to the enzymatic degradation with the mentioned defects. Several more micrographs of the samples have been shown in Fig. 2s of the Supplementary Materials.

### Weight loss and tensile strength measurements

The weight of samples was measured before and after the degradation process and the obtained results in terms of weight loss percentage have been reported in Table 1. There were no changes observed in the weight of unblended PE (B_α_ and B_s_) and untreated blended (A_0_) samples while a significant reduction was detected in PE-starch blend treated by the enzyme (A_α_). Interestingly, the weight reduction percent in the presence of alpha-amylase was more than the initial starch amount (25%) in each sample. This means that in addition to starch, polyethylene has been degraded in the system. In fact, there should be a cometabolic behavior in biodegradation of polyethylene in combination with enzymatic hydrolysis of starch. Similar results have been reported previously for biodegradation of starch-PE blend samples in a culturing medium of amylase-producing *Vibrios*^33^. The observations of Liu *et al*. through FTIR-microscope with ATR showed that the carbonyl groups formation mainly appeared around the starch granules in the samples which could be considered as an evidence for the positive role of starch on the polymer degradation^12^.

**Table 1:** Weight loss and tensile strength results of the samples after 31 days.

Sample	Weight loss (%)	Tensile strength (MPa)	Percent loss in tensile strength (%)
A_0_ (PE-Starch blend untreated)	0	2.17 ± 0.09	2
A_α_ (PE-Starch incubated with alpha-amylase)	48	0.27 ± 0.08	87
B_0_ (PE untreated)	0	11.35 ± 0.5	0
B_α_ (PE incubated with alpha-amylase)	0	11.0 ± 0.23	3

The starch content has inversely influenced the mechanical properties (tensile strength) of the PE-starch blend. This could be due to the existence of hydroxyl groups in the starch structure which are hydrophilic while on the contrary PE structure is hydrophobic in nature^14^. The minimum energy of the system could be achieved when the starch molecules take the V-type conformation which provides an interior hydrophobic space in the center and the hydroxyl groups form an outside hydrophilic surface^19^ (Fig. 3s, Supplementary Materials). The hydrophobic moieties such as PE can be replaced in the central space through the inclusion mechanism. This mechanism has been investigated for several starch-lipid or fatty acid systems which have nearly similar hydrophilic-hydrophobic conditions^19,20^. As a result, the crystalline portion of PE structure will be reduced after blending with starch leading to a lower tensile strength which is in agreement with previously reported results^9^. Aside from the PE degradation, the remarkable reduction of tensile strength for PE-starch blend after 31 days treatment can be explained by degrading of the starch in the samples. Partial biodegradation of starch can lead to the exposure of the hydrophobic polymer to the remained hydrophilic starch molecules causing a strong repulsion. This repulsion can be considered as the main cause for the formation of tiny cracks in the samples. To summarize the process, biodegradation of starch enfeebles the polymer matrix by creating porosity, craze, cracks, and loss of the integrity of polymer matrix which reduces the strength of the samples.

### Evaluation of biodegradation by thermal analysis

Differential scanning calorimetry (DSC) was used to measure the enthalpy of fusion, melting extrapolated onset temperature (T_eim_), melting peak temperature (T_pm_) and melting extrapolated end temperature (T_efm_) which indicates the degree of crystallinity and molecular weight range of the polymer in the samples. The results have been presented in Table 2.

**Table 2:** The enthalpy of fusion and three kinds of melting point temperature of PE-starch samples.

Sample	Incubation period (days)	∆H_m_ (J g^−1^)	T_eim_ (°C)	T_pm_ (°C)	T_efm_ (°C)
A_0_	—	−197.2	94	111.5	119
A_α_	31	−248.7	88	112	123

The more the enthalpy of fusion, the higher the degree of crystallinity^34^. The DSC profiles for a few selected samples have been shown in Fig. 4s of the Supplementary Material. The fusion enthalpy of PE-starch blends after treatment with alpha-amylase (A_α_) was obtained more than that of the untreated samples (A_0_). Accordingly, more crystallinity has occurred in samples after biodegradation process which is in agreement with the reported results^15,35^. In fact, LDPE is characterized by a high degree of short and long chain branching which reduces the crystallinity of the polymer structure. It is believed that branches are more susceptible to degradation which has been supported by the observations in this study as the enthalpy of fusion was higher for the samples after biodegradation. At the same time, melting peak temperature (T_pm_) and melting extrapolated end temperature (T_efm_) have increased which can be related to chain scission in side groups. In fact, LLDPE is produced by copolymerization of ethylene and higher alpha-olefins such as butene, hexene, or octene. Therefore, the number of carbon atoms in the side chains are more likely to be 2, 4 or 6. It is coming to be generally accepted that increasing the length of the side chains from 1 to 8 can make the molecular packing difficult and the increased flexibility of the side chains causes a reduction in the melting point^36^. Although, the heat of fusion of samples subjected to the enzymatic degradation was increased in comparison to the untreated samples, the melting extrapolated onset temperature (T_eim_) has reduced from 94 to 88 °C which in turn, reflects the reduction in the molecular weight of the polymer especially due to the failure in the main backbone of polyethylene chain. Therefore, the biodegradation seems to affect both the main backbone and the side chains of the polymer molecules. In order to have more perception of the effect of alpha-amylase on biodegradation of PE-starch blend, more analysis was carried out through FT-IR spectroscopy.

### Investigation of biodegradation by FT-IR

**Figure 2:**
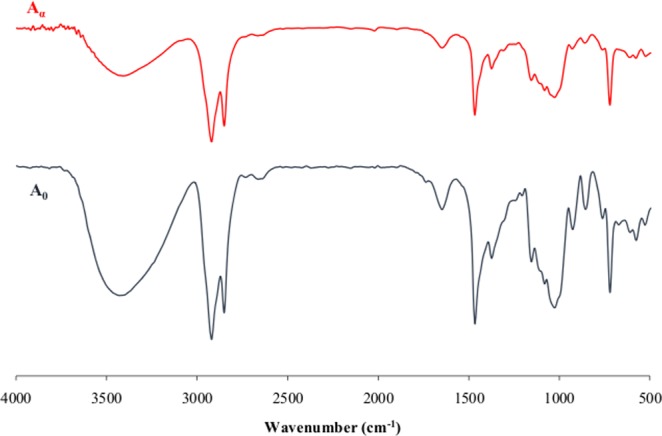
FT-IR spectra of PE-starch blends, A_0_: incubated in water, A_α_: incubated in the enzyme solution.

The modifications in the chemical structure of PE-starch samples were analyzed by studying their functional groups through FT-IR spectroscopy. The results for the blank and aqueous enzyme solution treated samples have been shown in Fig. 2. The O-H group in starch has a broad strong absorption in the region of 3400 cm^−1^. In polyethylene chain, the stretched C-H absorption occurs just before 3000 cm^−1^(2840–3000). Methylene and methyl groups have a bending absorption of about 1465 and 1375 cm^−1^ respectively. At 720 cm^−1^, the bending motion related to four or more CH_2_ groups in an open chain appears^37^. In comparison with untreated samples (A_0_) as the control, FT-IR spectra displayed a remarkable decrease in OH group in the presence of alpha-amylase (A_α_). Moreover, the intensity of peaks at 3000–2840 cm^−1^ in the treated samples (A_α_) decreased and at 1465 and 1375 cm^−1^, a notable reduction appeared. Further, a significant reduction in 720 cm^−1^ was observed in A_α_, which clarify the weakening of CH_2_ groups in the polymer chains. The results support our earlier claim that the polyethylene molecules have been degraded in combination with starch hydrolysis in the samples^25,38^.

### Energy dispersive x-ray spectroscopy (EDXS) analysis

The percentage of carbon and oxygen atoms as well as their weight percent for the control (A_0_) and enzymatically degraded samples (A_α_) have been calculated by EDS analysis and reported in Table 3. Elemental composition indicates that the percentage of carbon has decreased after the enzymatic treatment. In addition, the ratio of carbon atoms to oxygen atoms has reduced from 4.57 to 4.02

**Table 3:** The EDXS result of the control sample (A_0_) and enzymatically degraded sample (A_α_).

Sample	norm. Carbon [wt%]	norm. Oxygen [wt%]	norm. Gold [wt%]	Atom. Carbon [at%]	Atom. Oxygen [at%]	Atom. Gold [at%]	Atom C/Atom O	W C/W O
A_0_	75.80	22.09	2.11	81.94	17.92	0.14	4.57	3.43
A_α_	71.31	23.62	5.06	79.81	19.85	0.35	4.02	3.01

The ratio of carbon to oxygen atoms in the starch by the chemical formula of [(C_6_H_12_O_5_)_n_] is 6n/(5n + 1). For the starch with the highest degree of polymerization (n → ∞), this ratio approaches 1.2. The number of carbon atom of polyethylene [(CH_2_-CH_2_)_m_] is 2 m, so the ratio of carbon atoms of polyethylene to oxygen atoms in the starch in the A_0_ samples (untreated PE-Starch blend) can be calculated about 3.37 according to the following equations:

1 $${(\frac{{\rm{Atom}}{\rm{C}}}{{\rm{Atom}}{\rm{O}}})}_{0}={(\frac{{\rm{Atom}}{{\rm{C}}}_{{\rm{PE}}}+{\rm{Atom}}{{\rm{C}}}_{{\rm{St}}}}{{\rm{Atom}}{{\rm{O}}}_{{\rm{St}}}})}_{0}={(\frac{{\rm{Atom}}{{\rm{C}}}_{{\rm{PE}}}}{{\rm{Atom}}{{\rm{O}}}_{{\rm{St}}}})}_{0}+{(\frac{{\rm{Atom}}{{\rm{C}}}_{{\rm{St}}}}{{\rm{Atom}}{{\rm{O}}}_{{\rm{St}}}})}_{0}={(\frac{{\rm{Atom}}{{\rm{C}}}_{{\rm{PE}}}}{{\rm{Atom}}{{\rm{O}}}_{{\rm{St}}}})}_{0}+\frac{6{{\rm{n}}}_{0}}{5{{\rm{n}}}_{0}+1}\,$$

2 $${(\frac{AtomC}{AtomO})}_{0}={(\frac{Atom{C}_{PE}}{Atom{O}_{St}})}_{0}+1.2=4.57\to {(\frac{{\rm{Atom}}{{\rm{C}}}_{{\rm{PE}}}}{{\rm{Atom}}{{\rm{O}}}_{{\rm{St}}}})}_{0}=3.37$$

Similarly, the ratio of carbon to oxygen atoms in polyethylene and starch blend in A_α_ (PE-Starch incubated with alpha-amylase) can be determined by equation 3. It should be noted that if the starch were completely hydrolyzed to glucose after treatment with alpha-amylase, the ratio of carbon to oxygen atoms of the hydrolyzed starch would be reduced to 1. Therefore the ratio of the carbon to oxygen atoms for the hydrolyzed starch can be varied from 1 (for the completely hydrolyzed starch) to 1.2 (for the untreated starch).

3 $$\begin{array}{rcl}{(\frac{{\rm{Atom}}{\rm{C}}}{{\rm{Atom}}{\rm{O}}})}_{{\rm{\alpha }}} & = & {(\frac{{\rm{Atom}}{{\rm{C}}}_{{\rm{PE}}}+{\rm{Atom}}{{\rm{C}}}_{{\rm{St}}}}{{\rm{Atom}}{{\rm{O}}}_{{\rm{St}}}})}_{{\rm{\alpha }}}={(\frac{{\rm{Atom}}{{\rm{C}}}_{{\rm{PE}}}}{{\rm{Atom}}{{\rm{O}}}_{{\rm{St}}}})}_{{\rm{\alpha }}}+{(\frac{{\rm{Atom}}{{\rm{C}}}_{{\rm{St}}}}{{\rm{Atom}}{{\rm{O}}}_{{\rm{St}}}})}_{{\rm{\alpha }}}\\  & = & {(\frac{{\rm{Atom}}{{\rm{C}}}_{{\rm{PE}}}}{{\rm{Atom}}{{\rm{O}}}_{{\rm{St}}}})}_{{\rm{\alpha }}}+\frac{6{{\rm{n}}}_{{\rm{\alpha }}}}{5{{\rm{n}}}_{{\rm{\alpha }}}+1}={(\frac{{\rm{Atom}}{{\rm{C}}}_{{\rm{PE}}}}{{\rm{Atom}}{{\rm{O}}}_{{\rm{St}}}})}_{{\rm{\alpha }}}+(1\,{\rm{to}}\,1.2)\\  & = & 4.02\to {(\frac{{\rm{Atom}}{{\rm{C}}}_{{\rm{PE}}}}{{\rm{Atom}}{{\rm{O}}}_{{\rm{St}}}})}_{{\rm{\alpha }}}=(2.82\,{\rm{to}}\,3.02)\end{array}$$

The FT-IR results represent that starch has not been totally hydrolyzed and the ratio of carbon atoms of polyethylene to oxygen atoms of starch in the A_α_ sample can be estimated between 3.02 to 2.82. Consequently, the decrease of the mentioned ratio from 3.37 to 3.02–2.82 according to the EDXS analysis confirms that polyethylene deterioration has occurred alongside of the starch hydrolysis because the normalized number of the polyethylene carbon atoms has been dropped after the enzymatic treatment.

### Molecular weight measurements

**Figure 3:**
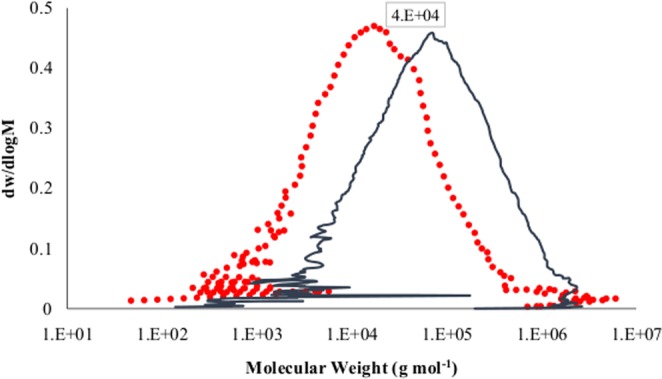
Distribution plot of HT-GPC of (−) the control sample and (•) enzymatically degraded sample.

The number average, peak average, weight average, z average and viscosity average molecular weights (M_n_, M_p_, M_w_, M_z_, and M_v_) of polyethylene for the control (A_0_) and enzymatically degraded samples (A_α_) have been reported in Table 4. Obviously, with reference to the control sample (A_0_), all types of the average molecular weights for the enzymatically degraded sample (A_α_) have decreased greatly as shown in Fig. 3. Apparently, some new shorter polymer chains with molecular weights less than 100 g mol^−1^ were formed in the PE-starch sample after the enzymatic degradation. On the other hand, the weight fraction of molecules having the molar mass less than 4 × 10^4^ has increased while the weight fraction of the heavier molecules has reduced. The dramatic plunge in molecular weight and formation of shorter chain lengths of polyethylene after enzyme treatment indicate the feasibility of the biodegradation of the synthetic polymer which has arisen from the scission event at the polyethylene backbone.

**Table 4:** The average molecular weights (g mol^−1^) of the control sample (A_0_) and enzymatically degraded sample (A_α_).

Sample	M_n_	M_p_	M_w_	M_z_	M_z+1_	M_v_	IV_n_	IV_p_	IV_w_	IV_z_
A_0_	19837.5	76990.2	151015	538584	1039540	94496.1	0.356015	0.509869	0.560231	0.813174
A_α_	5298.19	18552.2	46786	267819	613674	24784.4	0.146637	0.190043	0.213927	0.307954
Percent of decrease	73.29	75.9	69.01	50.27	40.96	73.77	58.81	62.72	61.81	38.53

To put it differently, the chromatography analysis results corroborated that alpha-amylase as the only biological agent in the system can promiscuously act on polyethylene-starch blend and cause a significant reduction in all types of PE molecular weight, particularly in M_n_, M_p_, and M_v_ with over 73% decrease. While amylase is considered as a specific enzyme for the starch hydrolysis, the promiscuous function of amylase which can be directly connected with its intrinsic aspect can influence the polyethylene^39^.

**Figure 4:**
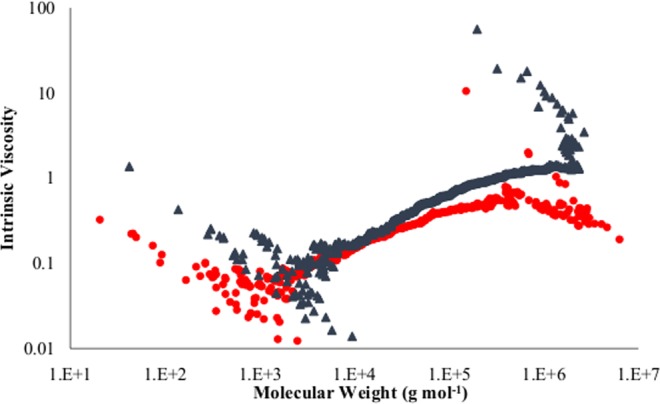
Intrinsic viscosity in terms of molecular weight of control sample (▲) and enzymatically degraded sample (•).

**Figure 5:**
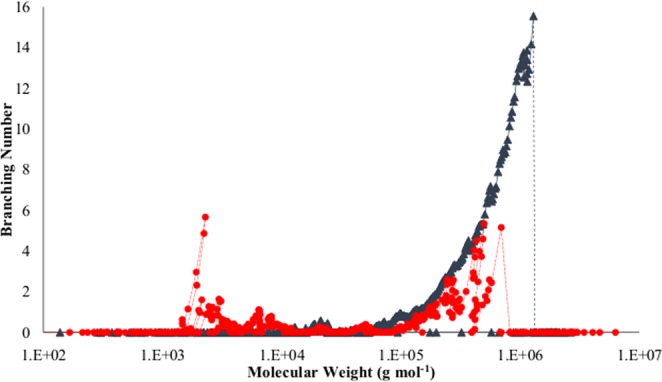
Branching number in terms of molecular weight of control sample (▲) and enzymatically degraded sample (•).

Moreover, number average, peak average, weight average and z average of the intrinsic viscosity (IVn, IVP, IVw, and IVz) of polyethylene in the control (A_0_) and enzymatically degraded samples (A_α_) were reported in Table 4. In comparison to the control sample (A_0_), a reduction in all types of intrinsic viscosities of the enzymatically degraded sample (A_α_) were achieved as demonstrated in Fig. 4. The obtained results implied that chain-scission at both the backbone and side branches of polyethylene chains has occurred. It is believed that there is a direct relation between the intrinsic viscosity of polymers and their chain length and branching number. As it has been shown in Fig. 4, in a similar molar mass, the intrinsic viscosity of the control sample (A_0_) was higher than the enzyme-treated sample (A_α_) which indicated that the branching number was smaller for the enzyme-treated sample (A_α_). The viscosity difference between the two samples was intensified for heavier fractions of the polymer for upper than 10^4^ g mol^−1^ molar masses. At the highest molecular weight extreme, the viscosity of the treated sample decreased to one-tenth of the control sample. Similarly, the branching number in the treated sample reduced significantly as can be seen in Fig. 5. Apparently, in the molar mass distribution between 10^5^ and 10^6^, the branching number of the control sample (A_0_) was higher than the treated polymer (A_α_) which demonstrated that the lateral chain branches of polyethylene were affected by the enzymatic degradation so intensive that all of the branches of some polymer chains have been entirely degraded.

### Estimation of changes in composition of PE-Starch sample after the enzymatic treatment process

**Figure 6:**
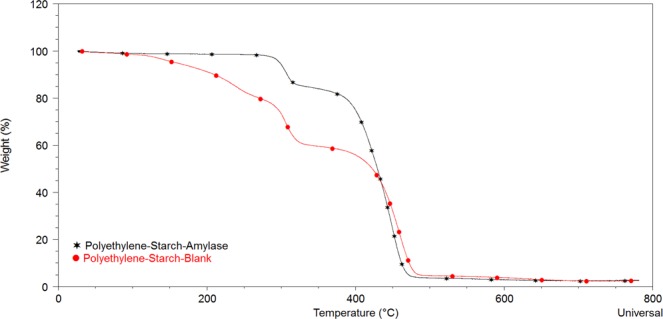
Classic tracing from the thermogravimetric analysis of control sample (•) and enzymatically degraded sample (*).

Thermal gravimetric analysis (TGA) was employed to analyze the mass changes of the samples as a function of the temperature rise. The percentages of the weight loss in a wide range of temperature variation (from 25 to 800 °C) under a constant heating rate for the control (A_0_) and enzymatically degraded samples (A_α_) have been reported in Table 5. In the temperature interval of 200 °C to 500 °C, a manifest weight loss was occurred in both samples with some variations. Generally, evaporation of the volatile fractions takes place in temperatures between 25 °C to 200 °C. The obtained results showed that the weight loss of the control sample (A_0_) was seven times higher than the enzyme treated sample in this temperature window which indicated that the highly volatile materials had been removed previously during the enzymatic treatment. In the range of 200 °C to 400 °C, the starch and glycerol content of the blend are mainly affected. The pyrolytic volatilization of the starch occurs in the temperature range of 250 °C to 350 °C and the normal boiling point of glycerol is 290 °C. Therefore, the higher the starch content, the greater the amount of mass changes through thermal gravimetric analysis in the mentioned temperature interval^40^. Seemingly, the enzyme treated sample (A_α_) had a smaller weight loss than the control (A_0_) because a portion of its starch content had been hydrolyzed by the enzyme. On the other hand, as it was shown in Fig. 6, the slope of the enzyme treated sample diagram has plunged in the temperature interval of 350 °C to 450 °C, so that the weight loss percentage of the treated sample (A_α_) was 32% higher than the control (A_0_). Evidently, the shorter polymer chains with lower molecular weights and naturally with lower pyrolytic volatile temperatures have been formed in the PE-starch sample after the enzymatic degradation which explains the mentioned observation. At higher temperatures (more than 500 °C), the majority of polymers have been evaporated and both of the samples showed a similar result. Obviously, the obtained TGA profiles in this section are in agreement with the earlier GPC results.

**Table 5:** The percent weight loss of the control sample (A_0_) and enzymatically degraded sample (A_α_) through TGA.

Sample	Temperature (°C)			
	25–200	200–400	400–600	600–800
Weight decrease(%) A_0_	8.795	35.29	52.11	0.2271
Weight decrease(%) Aα	1.231	23.82	71.96	1.326

The positive effect of blending PE with starch on its degradability has been known for a long time. Many researchers tried to justify this effect by physical and/or chemical explanations such as the creation of pores and cracks in the polymer and addition of active functional groups (e.g. carbonyl groups) to the PE structure. However, it has not been clarified that why would a fine powder of PE be still recalcitrant is spite of the large surface area or how can the functional groups be added to the PE molecules while the PE molecules are completely saturated and stable. The obtained results in this work showed that the partial hydrolysis of the V-type starch causes strong hydrophilic hydrophobic repulsions leading to cracks in the polymer structure. Beyond this, it was proved that amylase and starch were effective in degradation of PE molecules. In fact, amylase performs two separate but complementary activities in PE-starch blend degradation. First, the amylase decomposes the starch as its regular specific function and second as a promiscuous function, in the presence of starch breaks the PE molecules. Noticeably, alpha-amylase influenced the degradation of polyethylene indirectly in combination with starch hydrolysis in the blend of PE-Starch while it had no effect on the polyethylene samples without starch. Therefore, the degradation process can be considered as a cometabolic procedure where a complex of starch-amylase or a product of their reaction influences the PE breakdown. However, the exact mechanism has not been known yet and should be studied in detail in the future.

### Conclusions

Biodegradation of LDPE-starch blend samples by an alpha-amylase aqueous solution were studied. Tensile strengths of the samples after degradation process decreased significantly which was explained by the specific inclusive structure of the blend and the formation of tiny cracks in the matrix of samples because of the strong hydrophobic-hydrophilic interactions between the polymer and starch molecules after partial degradation of the starch. Moreover, the obtained results confirmed the breakdown of PE molecules at the presence of starch and amylase. The presented evidence showed that alpha-amylase in the presence of starch has a non-specific effect on polyethylene molecules. In other words, amylase has a cometabolic behavior in which it hydrolyzes the starch as the primary specific substrate and at the same time, its complex with the starch or a product of starch hydrolysis reaction decomposes the PE molecules. The observed influence of amylase on a non-specific polymeric substrate such as PE has been reported for the first time in this work.

## Materials and Methods

### Chemicals

Alpha-Amylase Liquozyme SC DS was Novozymes production. Glycerol was supplied by Guangzhou Wanjingfeng Company (S. Korea). LLDPE LL0209AA was from Bandar Imam Petrochemical Company (Iran). Na_2_HPO_4_ and NaH_2_PO_4_ were purchased from Merck. The nutrient broth was from Thermo scientific, and unmodified wheat starch was obtained from domestic suppliers (Gol Yas Mashhad-Iran).

### Starch blended polyethylene samples preparation

Starch is a natural polymer consist of amylose and amylopectin units. Amylose linear structure composes of crystalline and amorphous regions, and amylopectin can be added as branches on the linear part. In order to obtain a thermoplastic product with homogeneous properties, the amylose crystalline structured units can increase by the addition of glycerol as a plasticizer. Moreover, glycerol improves the processability and flexibility of the starch- polyethylene blend. Glycerol increases swelling in the PE-starch blend, thereby improving the water absorption which facilitates the enzyme diffusivity and therefore enhances the susceptibility of the blend^31^.

Two different series of PE blends (PE-Starch-Glycerol and PE-Glycerol at a weight ratio of 2/1/1 and 3/1) were prepared in a two-step procedure. Firstly, the components were weighed out carefully and added together in a mixer (Zhangjiagang Huaming Machinery Company, China). Then, sheets (thickness of 2 ± 0.1 mm) were produced from the blend using an injection machine under pressure of 20 MPa (Ningbo Jinhua plastic Machinery Industrial Company, China). The sheets were cut in a dumbbell-shape form according to ASTM D638-10. In addition, rectangular samples with 2 × 8 × 35 (mm^3^) dimensions were provided for physical testing.

### Enzymatic degradation

The enzymatic degradation tests were performed in triplicates at 25 °C and neutral pH (7.0 ± 0.5) using an alpha-amylase aqueous solution. The enzyme (Alpha-Amylase Liquozyme SC DS) activity was determined according to Fuwa method^41^ equal to 20.19 (U mL^−1^). It was added to distilled water in a 1/100 (v/v) ratio to produce the enzyme solution. Three dumbbell-shaped PE-starch blend samples were inserted in the enzyme solution and incubated for 31 days at the ambient temperature while the blank samples were put in the distilled water under the same conditions. After this time, degradation of the samples was analyzed by the relevant physical, mechanical, thermal and chromatography methods.

### Sample characterization and degradation analysis

The rectangular samples were oven-dried at 40 °C for 24 h before biodegradation test, and their initial weight was obtained (w_0_). At the end of the tests period, the samples were washed with distilled water and dried again at 40 °C for 24 h. Then, the final weight of the degraded samples (w_d_) was measured. The weight loss percentage of specimens after biodegradation was calculated by the following equation:

4 $${\rm{Weight}}\,{\rm{loss}}\,( \% )=[({W}_{0}-{W}_{d})/{W}_{0}]\times 100$$

The reported values are the average result of five samples treated under the same conditions.

The tensile strength was measured according to ASTM D882-12 using a tensile testing machine (Zwick/Roell Company, Germany) at 25 °C. The test speed was 5 mm min^−1^. Thermal properties of samples were determined using differential scanning calorimeter (TA instruments DSC (Netzsch 200F3 model, Germany)) in a nitrogen atmosphere as the purge gas according to ASTM D3418-12. 8 mg of the sample was sealed in aluminum pans and subjected to heating condition from 40 to 200 °C at a rate of 10 °C min^−1^. Three samples for each biodegradation test were analyzed. The DSC analysis provided the enthalpy of fusion and melting point of the polymer samples.

The functional groups of samples before and after degradation experiments were studied through FT-IR spectroscopy. The analytical test was run using FT-IR 6300 apparatus made by JASCO, Germany. FT-IR spectroscopy was carried out using spectral resolution of 1 cm^−1^ and scanning range from 400 to 4000 cm^−1^ in 25 °C according to ASTM D5477-11.

In addition, microscopic studies of samples were carried out based on the procedure of ASTM E986-04 with a TESCAN MIRA3 XMU VP-FESEM **(**Australia) scanning electron microscope (SEM) operating at 15 kV using the secondary electron detector (SE). Quantitative analysis of samples by energy dispersive spectroscopy (EDS) was also performed according to ASTM E1508-12.

Moreover, the polyethylene molecular weight and its distribution were analyzed by GPC (Agilent PL-220, USA) instrument according to ASTM D6474-12 to evaluate the degree of biodegradation. All measurements were recorded using a High-Temperature GPC (HTGPC) system equipped with differential refractive index (DRI), light scattering (LS) and viscometer (VS) detectors. GPC Analysis was performed using 3 PLgel 10 μm Mixed-B (300 × 7.5 mm) columns at 160 °C while 1,2,4-trichlorobenzene (TCB, 99%, ACROS Organics, Belgium, CAS 120-82-1, refractive index 1.5719) used as the solvent and the mobile phase at a flow rate of 1 mL min^−1^. The PE samples were dissolved to obtain concentrations of 1 mg mL^−1^. The calibration was also performed with Easivial PS-H (all three grades green, red and yellow) which their molecular weights were covering the whole range of 162 and 6000000 g mol^−1^.

The final test for confirmation of the irregular function of amylase in biodegradation of the PE-starch blend was thermogravimetric analysis that was accomplished by TGA instrument (TA Instruments Q500, USA) as stated in ASTM E1131-08. Nitrogen with purity of 99.9% was used as an inert compressed gas and reactive compressed gas was air. The flow rate and the ramp were adjusted to 90 mL min^−1^ and 10 °C min^−1^ respectively, and the range of the applied temperature was 25 to 800 °C.

## Supplementary information

Supplementary Materials

## Data Availability

All data generated or analyzed during this study are included in this published article (and its Supplementary Information files).
