# Supplementary material for: The promiscuous activity of alpha-amylase in biodegradation of low-density polyethylene in a polymer-starch blend
Source: Sci Rep. 2019 Feb 22;9:2612. doi: 10.1038/s41598-019-39366-0 (PMC6385501; doi:10.1038/s41598-019-39366-0)
Supplement: Supplementary file 1 — Supplementary Materials [file 41598_2019_39366_MOESM1_ESM.pdf]

Supplementary Materials:

The promiscuous activity of alpha-amylase in biodegradation of low-density polyethylene in a polymer-starch blend

M. Karimi[1], D. Biria[11]

1: Department of Biotechnology, Faculty of Advanced Sciences and Technologies, University of Isfahan, Isfahan, Iran

This file contains 5 figures.

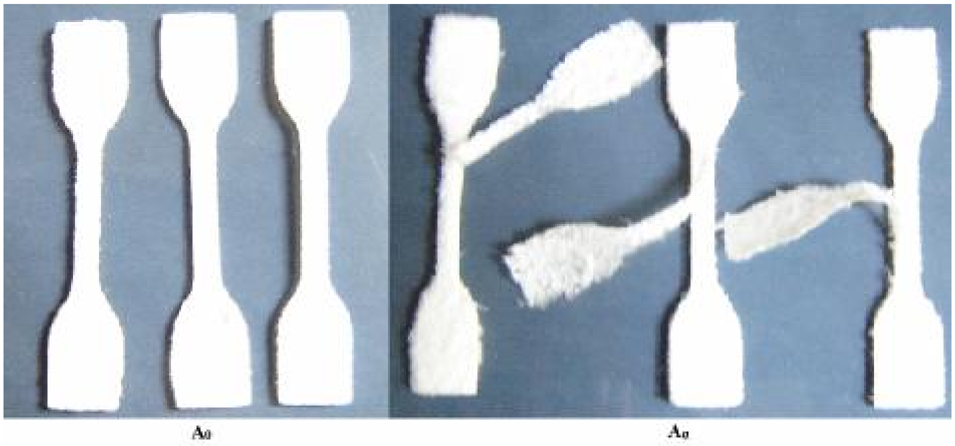

Fig. 1s: (A_0) PE-Starch incubated in water, ($A_α$) PE-Starch incubated with alpha-amylase

[1] Corresponding to D. Biria, E-mail address: d.biria@ast.ui.ac.ir, Tel: (98) 31 37934373

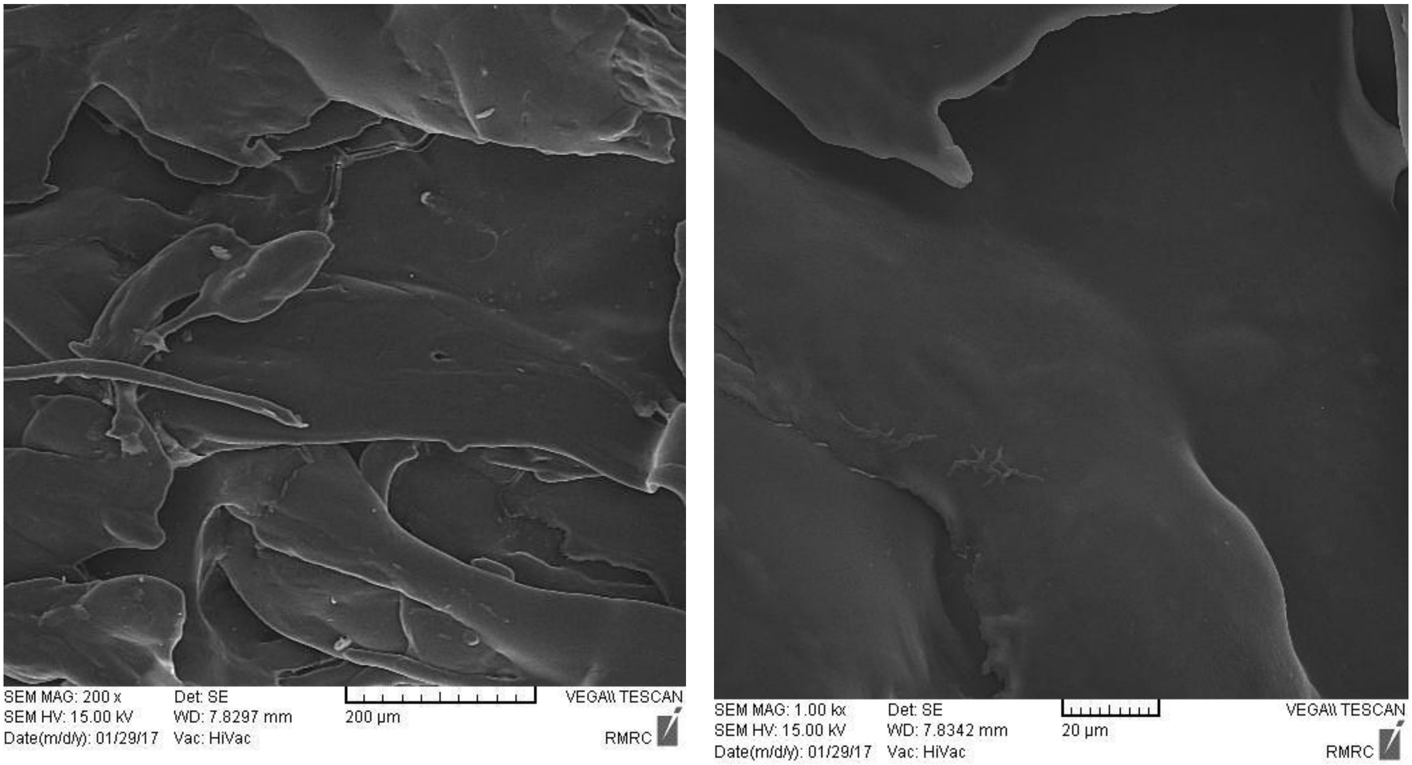

Fig. 2s: Scanning electron micrographs of PE-starch before (right) and after enzyme treatment (left)

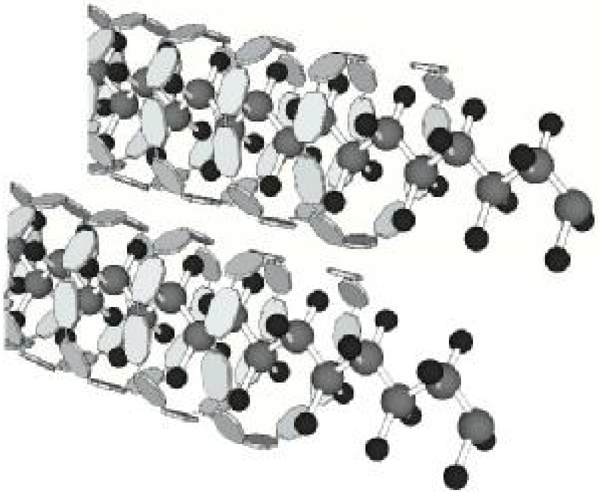

Fig. 3s: The proposed structure for the inclusion of polyethylene in V-type starch

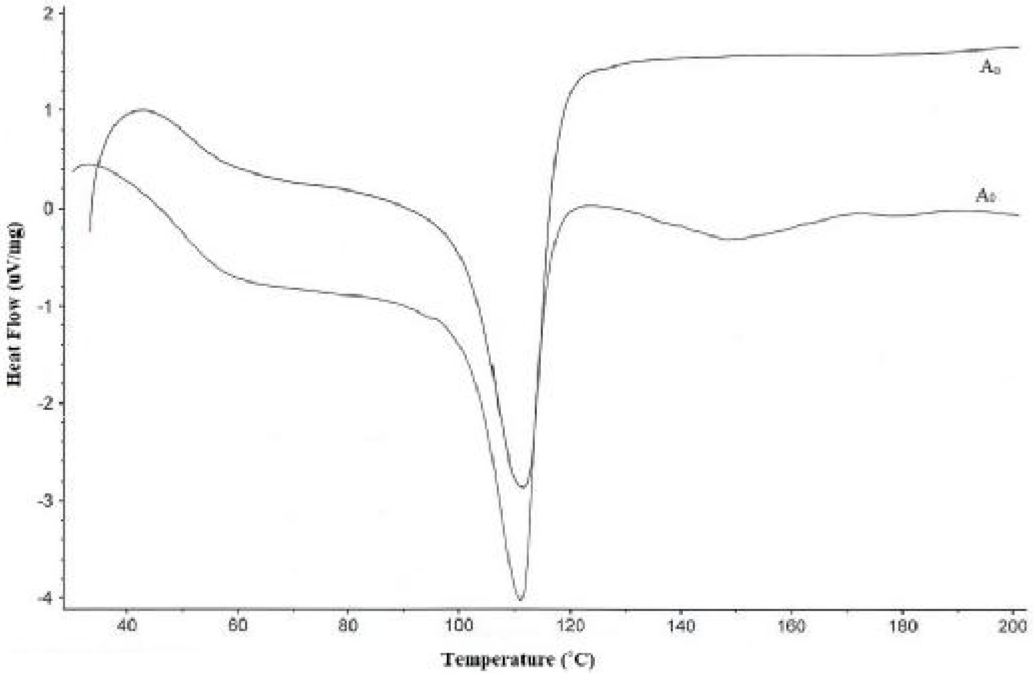

Fig. 4s: DSC profiles for PE-starch blends, A_0: incubated in water, A_α: incubated in the enzyme solution

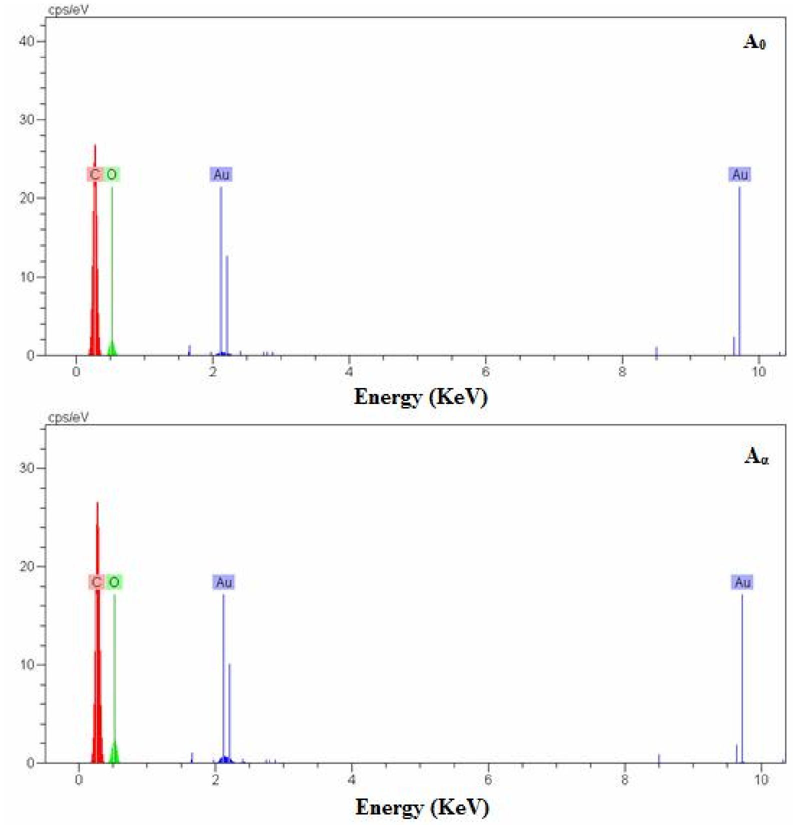

Fig 5s. EDS Diagrams
